# A simple technique to measure TAPSE and MAPSE on CMR and normal values

**DOI:** 10.1186/1532-429X-16-S1-P22

**Published:** 2014-01-16

**Authors:** Heerajnarain Bulluck, Hataichanok Ngamkasem, Daniel Sado, Thomas A Treibel, Marianna Fontana, Viviana Maestrini, James Moon, Derek J Hausenloy, Anna S Herrey, Steven K White

**Affiliations:** 1 The Hatter Cardiovascular Institute, London, UK; 2 Heart Hospital Imaging Center, Heart Hospital, University College London, London, UK

## Background

Mitral and Tricuspid Annular Plane Systolic Excursion (MAPSE, TAPSE) add valuable information to functional assessment of the right and left heart, highlighting abnormality even when ejection fraction is normal. To date, CMR-equivalents of MAPSE and TAPSE have been variably measured by a number of different methods but no formally published CMR guidelines on methodology, or normal values, exist. Further evaluation is required. The feasibility of a simple and quick technique is assessed in terms of its reproducibility, in order to provide age-stratified normal values.

## Methods

**Figure 1:**
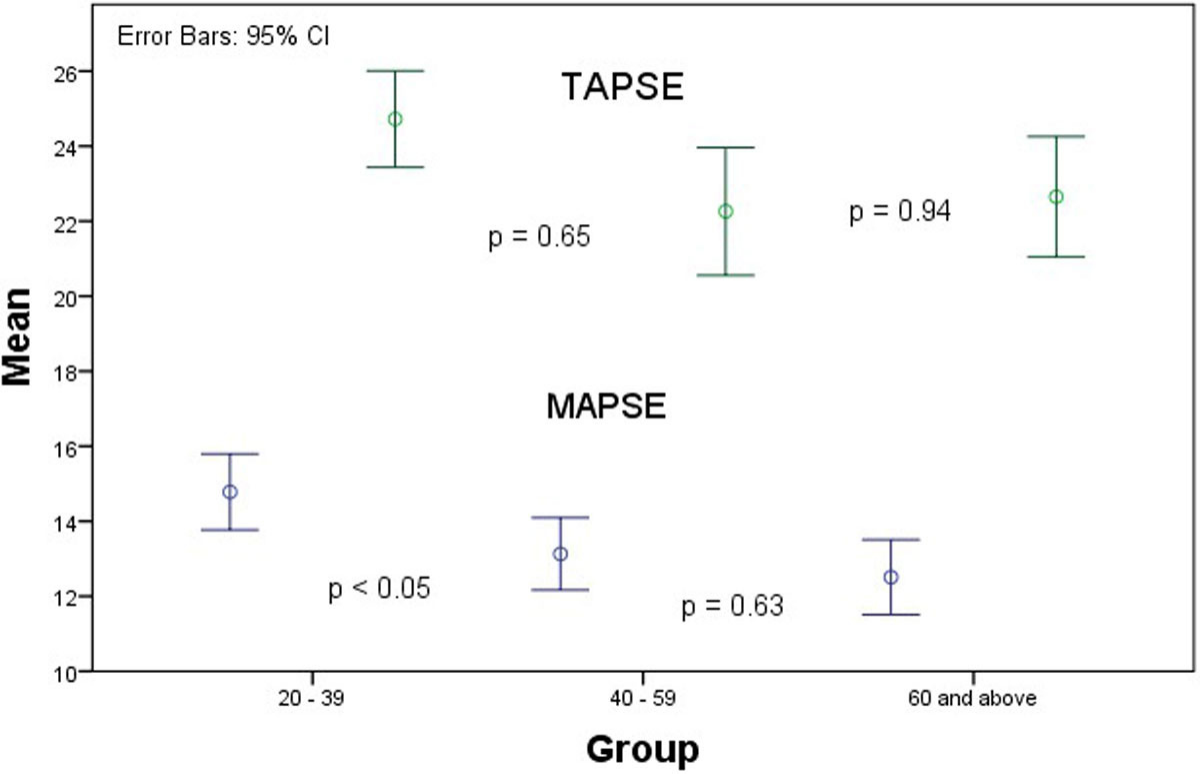
**Error Bars with 95% Confidence Interval for TAPSE (green) and MAPSE (blue) for the 3 age groups**.

Healthy volunteers (n = 68, 24 to 83 years-old, 32 males) were recruited from local advertisements. CMR-equivalent MAPSE and TAPSE were measured on standard 4-chamber SSFP cine images, independently by 2 blinded observers. The straight-line distance travelled by the lateral tricuspid and mitral annulus from end-diastole to end-systole was measured as shown in Figure 1. Inter-observer variability and intra-observer variability were calculated in a sample (n = 20) of subjects. Normal values were then grouped according to age: group 1: 20-39 yrs (n = 25); group 2: 40-59 yrs (n = 21) and group 3: ≥ 60 yrs (n = 22).

## Results

**Figure 2:**
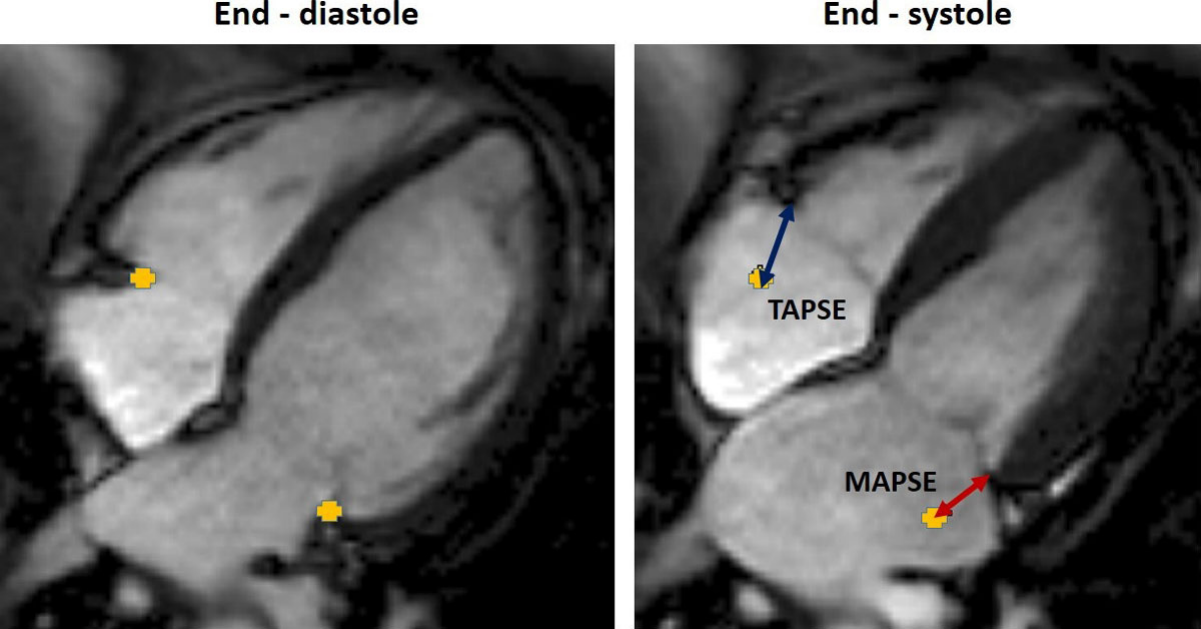
**The distance travelled by the lateral annulus from end-diastole to end-systole are measured to obtain CMR-equivalent of TAPSE and MAPSE**.

There was good inter-observer agreement for MAPSE (Intraclass Correlation Coefficient (ICC) of 0.84, 95% CI 0.60 - 0.94, p < 0.001) and TAPSE (ICC 0.77, 95% CI 0.43 - 0.91, p < 0.05). There was also very good intra-observer agreement (ICC for MAPSE: 0.84, 95% CI 0.64 - 0.93, ICCC for TAPSE: 0.92, 95% CI 0.80 - 0.97, p < 0.001). There were no significant differences in MAPSE and TAPSE between genders. Subgroup analysis showed no difference in TAPSE in the 3 different age groups. MAPSE was different between groups 1 and 2 and but there was no statistical difference between groups 2 and 3 (Figure 2). Therefore, from our cohort of healthy individuals, TAPSE was 23.3 ± 3.6 mm for the whole cohort. MAPSE was 14.8 ± 2.4 mm for those between 20 to 39 years of age and 12.8 ± 2.2 mm for those aged 40 years old and above.

## Conclusions

MAPSE and TAPSE are easily measured, highly reproducible CMR parameters. Although these parameters should not replace formal volumetric evaluation of systolic function, they add value in patients with impaired left ventricular function despite normal EF (e.g. hypertrophic cardiomyopathy), complement our assessment of right ventricle function, and provide a platform to determine their clinical importance.

## Funding

Dr Bulluck is employed by The Hatter Cardiovascular Institute. This work was undertaken at UCLH/UCL and a proportion of the funding came from the Department of Health National Institute for Health Research Biomedical Research Centres funding scheme.

